# Addition of high C:N crop residues to a P-limited substrate constrains the benefits of arbuscular mycorrhizal symbiosis for wheat P and N nutrition

**DOI:** 10.1007/s00572-021-01031-8

**Published:** 2021-04-23

**Authors:** Rosolino Ingraffia, Sergio Saia, Antonio Giovino, Gaetano Amato, Giuseppe Badagliacca, Dario Giambalvo, Federico Martinelli, Paolo Ruisi, Alfonso S. Frenda

**Affiliations:** 1 Dipartimento di Scienze Agrarie, Alimentari e Forestali, Università degli Studi di Palermo, Viale delle Scienze, 90128 Palermo, Italy; 2 Department of Veterinary Sciences, University of Pisa, Via delle Piagge 2, 56124 Pisa, Italy; 3 Council for Agricultural Research and Economics, Research Centre for Plant Protection and Certification (CREA-DC), SS 113 km 245.500, 90011 Bagheria (PA), Italy; 4 Dipartimento di Agraria, Università Mediterranea di Reggio Calabria, Feo di Vito, 89124 Reggio Calabria, Italy; 5 Dipartimento di Biologia, Università degli Studi di Firenze, Via Madonna del Piano 6, 50019 Sesto Fiorentino (FI), Italy

**Keywords:** *Triticum durum*, Plant growth, ^15^N fertiliser recovery, N:P ratio, Canonical discriminant analysis, Pot experiment

## Abstract

Many aspects concerning the role of arbuscular mycorrhizal (AM) fungi in plant nutrient uptake from organic sources remain unclear. Here, we investigated the contribution of AM symbiosis to N and P uptake by durum wheat after the addition of a high C:N biomass to a P-limited soil. Plants were grown in pots in the presence or absence of a multispecies AM inoculum, with (Org) or without (Ctr) the addition of ^15^N-labelled organic matter (OM). A further treatment, in which ^15^N was applied in mineral form (Ctr+N) in the same amount as that supplied in the Org treatment, was also included. Inoculation with AM had positive effects on plant growth in both control treatments (Ctr and Ctr+N), mainly linked to an increase in plant P uptake. The addition of OM, increasing the P available in the soil for the plants, resulted in a marked decrease in the contribution of AM symbiosis to plant growth and nutrient uptake, although the percentage of mycorrhization was higher in the Org treatment than in the controls. In addition, mycorrhization drastically reduced the recovery of ^15^N from the OM added to the soil whereas it slightly increased the N recovery from the mineral fertiliser. This suggests that plants and AM fungi probably exert a differential competition for different sources of N available in the soil. On the whole, our results provide a contribution to a better understanding of the conditions under which AM fungi can play an effective role in mitigating the negative effects of nutritional stresses in plants.

**Supplementary Information:**

The online version contains supplementary material available at 10.1007/s00572-021-01031-8.

## Introduction

The majority of terrestrial plant species, including many agricultural crops, can form mutualistic associations with arbuscular mycorrhizal (AM) fungi (Smith and Read 2008) belonging to the subphylum Glomeromycotina (Spatafora et al. 2017). The potential benefits of AM symbiosis on plant growth and productivity are well known and include enhanced mineral nutrition and increased tolerance to both biotic and abiotic stresses (Pozo and Azcón-Aguilar 2007; Smith and Smith 2011a; Lenoir et al. 2016).

The main impact of AM symbiosis on plant nutrition and growth has been ascribed to an advantage in the uptake of low mobility ions, mainly phosphorus (P) (Li et al. 2006). According to Richardson et al. (2009) and Smith and Smith (2012), this may occur through different pathways: (i) increase in the volume of soil explored by the extensive extraradical hyphal network (that allows exploitation of soil beyond the depletion zone in the rhizosphere), (ii) higher substrate affinity of P uptake into fungal hyphae than directly into plant roots (due to the presence in the extraradical mycelium of high-affinity P transporters that allow mycorrhizal plants to acquire orthophosphate from soil solution at lower concentrations than roots alone; Benedetto et al. 2005), (iii) acidification of the mycorrhizosphere (that can increase the mobilisation of sparingly-soluble inorganic P compounds, although this occurs typically in alkaline soils), and (iv) possible fungal hydrolysis of organic P, especially under mineral P deficiency (Sato et al. 2015; Ezawa and Saito 2018; Andrino et al. 2019). Indirect effects of AM fungi on the availability of P to plants may occur through the alteration of the soil microbial community composition in the mycorrhizosphere (Barea et al. 2005) and, in particular, through the stimulation of AM-associated saprotrophic microorganisms (Hodge et al. 2010; Jansa et al. 2013).

In addition to the benefits related to P acquisition, the AM fungi also can play an important role in the plant’s uptake of nitrogen (N) (Hawkins et al. 2000; Hodge and Fitter 2010; Hodge and Storer 2015). Studies using ^15^N tracer techniques have revealed that AM hyphae can transfer N from the soil to host plant roots (Mäder et al. 2000; Tanaka and Yano 2005). Furthermore, a number of studies have shown that AM fungi may regulate the expression of many plant N transporter genes (Duan et al. 2015; Saia et al. 2015), thus suggesting their active role in this process.

Conditions under which the benefits described above materialise for both P and N have been widely investigated at various levels (molecular, biochemical, physiological, morphological, agronomic) and scales (cell, organ, whole plant, field, natural or agro-ecosystem) (Bucher 2007; Barea et al. 2008; Smith and Smith 2011b; Smith et al. 2011, 2018). However, the consensus on nutrient uptake by AM fungi and delivery to the plant remains under debate. In particular, the results available are highly variable depending on the plant species and its ability to transfer carbon (C) to the fungus, and on the AM fungus species (Duan et al. 2015; Fabiańska et al. 2019; Li et al. 2019; Teutscherova et al. 2019; Wipf et al. 2019), despite there being evidence that the plant may modulate the C transfer to the AM fungus (and thus, potentially the net benefit of the AM symbiosis) depending on either the lack of N or P, with a higher ability to exchange C for P than C for N when both P and N are limiting (Li et al. 2019).

Much research has shown that AM fungi can improve plant growth by enhancing nutrient capture from organic materials added to the soil (Saia et al. 2014; Thirkell et al. 2016; Bukovská et al. 2018). Furthermore, it also has been demonstrated that this effect may vary considerably depending on the physico-chemical characteristics of the OM added (e.g. form, C:N ratio, lignin content, quantity and quality of nutrients; Hodge et al. 2000a; Ingraffia et al. 2020; see also Hodge 2014). The addition of different organic materials can indeed affect in different ways the physico-chemical characteristics of the soil, the availability of nutrients, and the size and structure of the microbial community, which are all factors that can impact AM symbiotic functioning. Moreover, it has been reported that the effects of the addition of an organic material to a soil may differ depending on the “context” in which this occurs, particularly on soil characteristics, temperatures, and water availability.

In the cropping systems of the Mediterranean region, OM addition to the soil is represented almost exclusively by returns of crop residues (often of cereals, with a high C:N ratio) and this, in the absence of N fertilisation, frequently determines detrimental effects on plant growth because of temporary immobilisation of N by decomposers. Considering that in these environments P often is deficient in soils (or is present in forms that cannot be directly utilised by plants), under these circumstances, the AM symbiosis can provide plants with both advantages (by contributing to improve plant P uptake) and disadvantages (by entering the AM fungi in competition with plants for soil N; Püschel et al. 2016; Ingraffia et al. 2020). However, no information on this potential conflict is currently available. Therefore, we conducted an experiment on durum wheat (*Triticum durum* Desf.) to examine whether inoculation with AM fungi can enhance plant P and N uptake when crop residues with a high C:N ratio are added to a P-limited soil. We hypothesised that under these circumstances, the benefit of the AM fungi to plant P uptake could counterbalance or even outweigh any detrimental effects due to the reduction of N availability by favouring plant growth and indirectly also the plant N uptake. We grew wheat plants in pots in both the absence or presence of an AM fungal inoculum, and with or without the addition of crop residues with a high C:N ratio (biomass of oat, *Avena sativa* L.) to the soil. Additionally, a treatment in which N was applied in mineral form in an amount equivalent to the total amount contained in the added OM was included to avoid confounding effects on plant P uptake as a consequence of the possible increase in N availability due to the addition of OM. To trace the fate of the applied N and analyse the impact of the AM symbiosis on plant N uptake, both the crop residues and the mineral fertiliser were labelled with the ^15^N isotope.

## Materials and methods

The experiment was conducted at the Pietranera experimental farm (37° 30′ N, 13° 31′ E, 178 m a.s.l., Sicily, Italy). A completely randomised factorial design replicated four times was adopted. The treatments were (i) “mycorrhization”: inoculation with AM fungi (+AM) and non-inoculated control (−AM) and (ii) “fertilisation”: addition of OM (Org), non-fertilised control (Ctr), N fertilised control (Ctr+N).

Durum wheat plants were grown in pots (diameter 20 cm, height 50 cm), each filled with 13 kg of a soil:perlite mixture (70:30 v/v). The perlite (Perlite Italiana, Corsico, Milano, Italy) had a particle size of 1–2 mm. Agricultural soil was collected from the first 30 cm of a well-structured clay soil classified as a Vertic Haploxerept with the following properties: 242 g kg^−1^ clay, 235 g kg^−1^ silt, and 523 g kg^−1^ sand; pH 8.0 (1:2.5 H_2_O); 9.2 g kg^−1^ total C (Walkley–Black); 1.03 g kg^−1^ total N (Kjeldahl); and 1.90 dS m^−1^ saturated electrical conductivity (at 25 °C); 7 mg kg^−1^ extractable P (Olsen), and 135 mg kg^−1^ exchangeable K_2_O. The soil was sieved through a 2-mm mesh, and both the soil and perlite were heat-sterilised at 125 °C for 72 h. Sterilisation was performed in an aluminium bowl in which a layer of 1 cm water was added below 5 cm of sieved soil. This likely removed most of the mineral N via volatilisation. Before starting the experiment, each pot received 80 ml of soil suspension filtrate to reintroduce the natural microbial community excluding AM fungi. The natural microflora was extracted by suspending 1000 g soil in 4.0-L distilled water (shaken for 20 min at 140 swings per minute). After shaking and decanting, the suspension was filtered (16-μm mesh) to remove AM fungi.

A mix of eight AM fungus species (*Gigaspora margarita*, *Glomus aggregatum*, *Rhizophagus intraradices*, *Funneliformis mosseae*, *Glomus clarum*, *Glomus monosporum*, *Glomus brasilianum*, and *Glomus deserticola*) (Micronised Endo Mycorrhizae®, Symbio, Wormley, Surrey, UK) at a rate of 10 g per pot was used as inoculum. Total AM fungal spore density was 200 spores g^−1^ of inoculum by our check. Species composition was not assessed in our lab. The inoculum comprised 95% AM spores and 5% organic material as reported by the manufacturer. In the non-inoculated pots, no mock (sterilised) inoculum was added; however, the amount of OM added through the inoculum was extremely small, representing 0.25% of the total OM present in the substrate and about 1% of that added with the crop residues. The inoculum was thoroughly mixed within a 10-cm-thick layer of the mixed substrate; then, a layer of 3 cm of substrate was added and seeds were sown in the upper 3-cm layer.

The stable isotope ^15^N was used as tracer to follow the fate of applied N (through addition of OM or mineral N fertiliser) and to examine the impact of the AM symbiosis on plant N acquisition. For the Org treatment, a total amount of 40.3 g dry weight of a ^15^N-enriched biomass of oats with an isotopic enrichment of 1.02 atom % was added to each pot. This ^15^N-labelled OM was obtained by cultivation of oats during the previous cropping season in plots fertilised after complete crop emergence with 10 kg ha^−1^ of ammonium sulphate with an isotopic enrichment of 10 atom %. Oat plants then were collected at grain maturity, and the oat mature shoots (which in practice represent surface residues, i.e. straw plus stubble) were used in our experiment as organic material after oven-drying. The oat biomass was chopped by hand with a multiple blade shredder scissors and then with a mezzaluna knife; the cut material was passed through a 2-mm sieve. The fraction ≤ 2 mm was homogeneously distributed at a depth of 5–10 cm 1 day before sowing. The P and N concentrations of the oat biomass were 1.2 and 5.2 g kg^−1^ respectively, and the C:N ratio was approximately 80:1. For the Ctr+N treatment, an equivalent amount of N compared to that in the Org treatment was applied; so each pot received a total amount of 1 g of a ^15^N-fertiliser as ammonium sulphate ([NH_4_]_2_SO_4_) with an isotopic enrichment of 10 atom %. The total N fertiliser for each pot was divided into three equal amounts and applied at 24, 46, and 66 days after sowing.

Twenty-five seeds of durum wheat (cv. Anco Marzio), previously surface-sterilised with H_2_O_2_ at 4% for 3 min, were sown in each pot. Ten days after emergence, plants were thinned to 14 seedlings per pot. All pots were placed outdoors and covered by a transparent roof to protect them against precipitation. The temperature regime during the experimental period is shown in Fig. S1. The water holding capacity of the substrate was determined with the gravimetric method (Dobriyal et al. 2018). Briefly, 10 perforated crucibles were filled with 100 g substrate and placed in a basin with water up to half of the height of the crucibles. The crucibles were allowed to absorb water by capillarity until each was saturated. Excess water was allowed to drain, and the crucibles were weighed and then oven-dried at 105 °C to a constant weight. The difference in weight between the crucibles before and after the drying process represented the soil water content at field capacity. During the experiment, irrigation was done using tap water (0.58 dS m^−1^ electrical conductivity at 25 °C) weekly and, for each pot, the amount of irrigation water consisted of total replenishment of water lost through evapotranspiration. Evapotranspiration losses were determined by measuring pot weight before each irrigation event. In this way, leaching was avoided and soil water was maintained above 70% of the water holding capacity.

All pots were harvested 91 days after sowing, at the beginning of anthesis (stage 60, Zadoks scale; Zadoks et al. 1974). The plant biomass was immediately separated into shoots and roots, and fresh weights were recorded. Roots were gently separated and washed several times in tap water until clean. Afterwards, they were carefully dried with paper towels. A representative root sample (about 1 g dry weight, corresponding to around 5 g fresh weight) was taken from each pot to determine the overall colonisation of roots by AM fungi. To take the representative root sample, subsamples of the root systems were taken from several roots in five positions: close to the upper part the root system, close to the root tips, and in three middle points. These subsamples were mixed and placed in a biopsy cellette rapidly saved in cold water (around 5 °C) for 1 day. Root samples were cleared with 100 g L^−1^ potassium hydroxide (KOH) and stained with 50 mg L^−1^ trypan blue following the method described by Phillips and Hayman (1970) without the use of phenol (Vierheilig et al. 2005). Root colonisation by AM fungi was then measured with the grid intersect method (Giovannetti and Mosse 1980) counting 280 intersections per sample at 10 × magnification under a dissecting microscope. For each pot, the remaining plant biomass was dried at 65 °C for 36 h to determine the belowground and aboveground dry weights. Shoot N and ^15^N concentrations were determined using an isotope ratio mass spectrometer (20–20 interfaced to a Roboprep-CN, Europa Scientific Ltd, Crewe, UK). Shoot P concentrations were determined using the method described by Bertramson (1942), after turning dry mass to ash (at 550 °C for 48 h) and without the addition of magnesium nitrate.

Root length was estimated from the number of intersections of a sample of roots with a grid according to Tennant (1975). Each sample analysed had a mean dry weight of 66.3 mg (range: 39.8–136.9 mg). On each sample we counted on average 356 intersections (range 178–630) which provided each a mean specific root length of 56 m g^−1^ (range 44–72). The conversion factor of the gridded plate used for the root length compared to Tennant’s (1975) measures was 1.07927. Following root length and dry matter determinations, specific root lengths (SRL, m root g^−1^ root) were calculated.

Data on ^15^N enrichment of biomass were used to calculate labelled-fertiliser N recovery (^15^*N*_REC_) on a pot basis (g N pot^−1^) and on a percentage basis according to Hauck and Bremner (1976):

1 $${{}^{15}N}_{\mathrm{REC}}=N_t\times\frac{{}^{15}N_{\mathrm{fp}}-{}^{15}N_{\mathrm{nfp}}}{{}^{15}N_{\mathrm{fert}}-{}^{15}N_{\mathrm{nfp}}}$$

and

2 $$\%{}^{15}N_{\mathrm{REC}}=\frac{{}^{15}N_{\mathrm{REC}}}f\times100$$

where *N*_*t*_ is the plant N content (g pot^−1^), ^15^*N*_fp_ is the atom % ^15^N in the fertilised plants (i.e. Ctr+N and Org treatments), ^15^*N*_nfp_ is the atom % ^15^N in the non-fertilised plants (i.e. the Ctr treatment), ^15^*N*_fert_ is the atom % ^15^N in the Ctr+N or the Org treatments, and *f* is the rate (g N pot^−1^) of the N fertiliser or the OM amendment.

### Statistical analysis

Because the AM fungal colonisation in the −AM treatment was negligible, statistical analysis on this trait was performed only on the +AM treatment using a one-way analysis of variance (ANOVA) to determine the effects of the fertilisation treatments. A two-way factorial ANOVA was used to determine the effects of the fertilisation and mycorrhization treatments, and of their interaction. The analyses were performed with R version 4.0.2 (R Development Core Team 2018). Shapiro and Bartlett tests were used to assess normality and homoscedasticity, respectively, of the model residuals. The response variable N concentration did not fulfil the ANOVA assumptions; therefore, data of this response variable were log-transformed. Following the ANOVA, pairwise comparisons (i.e. LSMEANS) using the ‘emmeans’ package (Lenth et al. 2020) and confidence intervals using the ‘dabestr’ package (Ho et al. 2019) were used to investigate the effects of mycorrhization within each fertilisation condition. All *p*-values derived from selected pairwise comparisons and confidence intervals of the differences are reported in tables and figures, as recommended by Gardner and Altman (1986). This method was used to avoid the problem of *p*-value dichotomous cut-offs (Wasserstein and Lazar 2016; Wasserstein et al. 2019). Non-transformed data are reported in figures. The ‘tidyverse’ package (Wickham 2017) was used to represent the data graphically.

Additionally, a canonical discriminant analysis (CDA) was carried out to separate the six treatment groups (with each group deriving from a single combination of mycorrhization × fertilisation) and to identify which of the measured traits contributed the most to distinguish these groups. CDA effectively projects the data into the space of linear combinations of the original quantitative variables that account for the greatest proportion of the among-group variance relative to within-group variance. Canonical variable means (centroids) were calculated for each group, and the significance between pairs of centroids was determined using the Mahalanobis distance at the 0.05% probability level. CDA was run using the data for all measured traits except for root AM colonisation and ^15^N recovery.

## Results

### Root mycorrhizal colonisation and plant growth

**Fig. 1:**
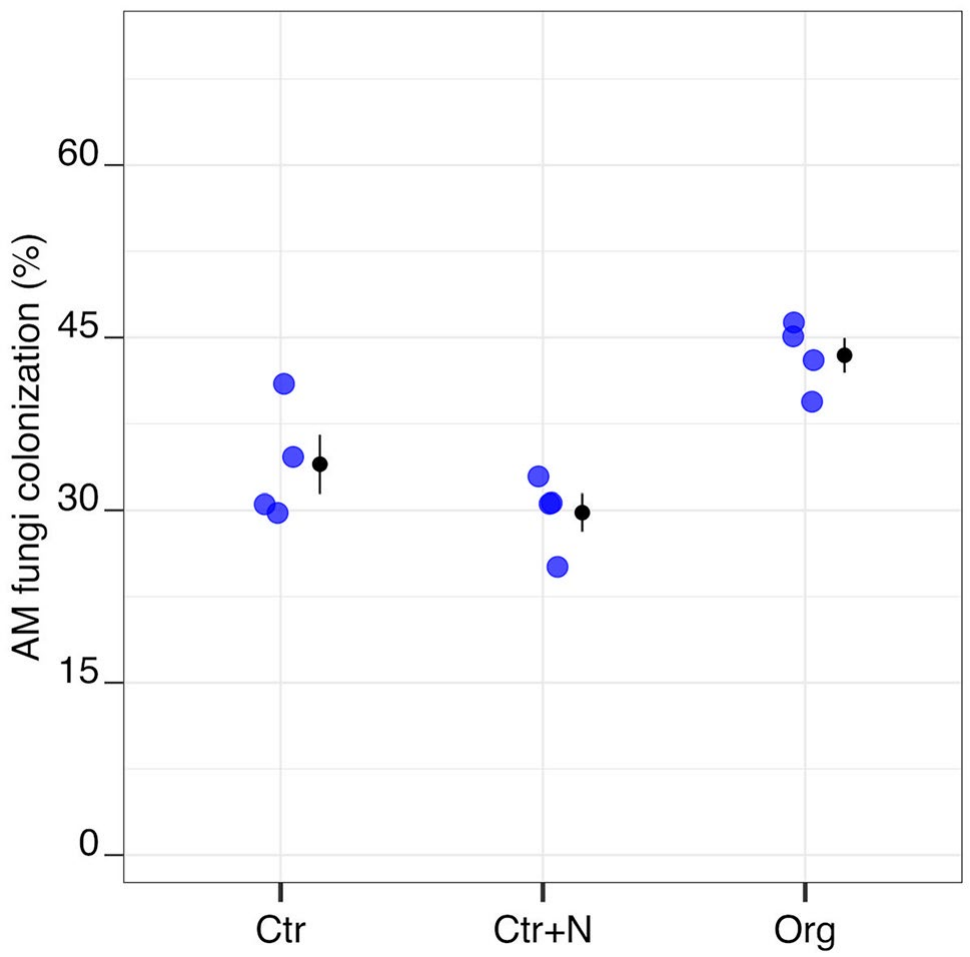
Arbuscular mycorrhizal (AM) fungi root colonisation of durum wheat in the different fertilisation treatments (Ctr control not fertilised, Ctr+N control fertilised with ammonium sulphate, Org soil amended with crop residues). Raw data are shown in the plot. Data are plotted with the mean depicted as a black circle ± SE (*n* = 4) represented by the end of the vertical black line to the right of the raw data

Non-inoculated (−AM) plants showed negligible levels of mycorrhizal colonisation (always < 0.5% of root length colonised). Characteristic structures of AM fungi were observed in the roots of the inoculated (+AM) plants; the levels of mycorrhizal colonisation differed with fertilisation treatment (Table 1), being higher in plants grown in the soil amended with crop residues (Org) than in plants grown under both the mineral N fertilised (Ctr+N) and non-fertilised control (Ctr) treatments (Fig. 1; Table 2).

**Table 1:** Analysis of variance: *F*- and *p*-values for the effects of the applied treatments (Fertilisation, Fert (1); Mycorrhization, Myc (2); and their interaction (1 × 2)) on the traits observed in durum wheat plants

	Fert (1)			Myc (2)			1 × 2		
	*df*	*F*-values	*p*-values	*df*	*F-*values	*p-*values	*df*	*F-*values	*p-*values
AM fungi colonisation	2	24.46	0.001	-	-	-	-	-	-
Aboveground biomass	2	12.58	< 0.001	1	17.13	< 0.001	2	0.53	0.601
Belowground biomass	2	39.85	< 0.001	1	9.37	0.008	2	0.73	0.497
Nitrogen concentration	2	73.66	< 0.001	1	2.23	0.116	2	1.45	0.261
Nitrogen content	2	175.18	< 0.001	1	24.21	< 0.001	2	14.80	< 0.001
^15^Nitrogen recovery	1	2915.12	< 0.001	1	0.61	0.454	1	14.77	0.004
Phosphorus concentration	2	0.37	0.693	1	64.77	< 0.001	2	11.21	< 0.001
Phosphorus content	2	7.21	0.007	1	65.79	< 0.001	2	7.00	0.007
N:P	2	42.95	< 0.001	1	49.12	< 0.001	2	4.48	0.030
Root length	2	40.56	< 0.001	1	12.67	0.003	2	1.54	0.246
Specific root length	2	7.15	0.007	1	6.45	0.023	2	2.70	0.099

**Table 2:** *p*-values for pairwise comparisons, effect size mean (unpaired means), and estimated 95% confidence intervals (Δ mean and CIs; in brackets) in the different fertilisation treatments (Ctr, control not fertilised; Ctr+N, control fertilised with ammonium sulphate; Org, soil amended with crop residues) in the absence (−AM) or presence (+AM) of arbuscular mycorrhizal fungal inoculum

	–AM						+AM					
	Ctr vs Ctr+N		Ctr vs Org		Ctr+N vs Org		Ctr vs Ctr+N		Ctr vs Org		Ctr+N vs Org	
	*p*-values	Δ mean ± 95% CIs	*p*-values	Δ mean ± 95% CIs	*p*-values	Δ mean ± 95% CIs	*p*-values	Δ mean ± 95% CIs	*p*-values	Δ mean ± 95% CIs	*p*-values	Δ mean ± 95% CIs
AM fungi colonization	-	-	-	-	-	-	0.173	−4.18 (−10.5; 0)	0.008	9.48 (3.41; 13.7)	0.001	13.7 (9.98; 17.6)
Aboveground biomass	0.207	0.23 (0.17; 0.31)	0.002	0.55 (0.19; 0.74)	0.069	0.31 (−0.06; 0.52)	0.263	0.21 (−0.08; 0.5)	0.029	0.37 (0.1; 0.65)	0.444	0.16 (0.04; 0.27)
Belowground biomass	0.088	−0.13 (−0.19; −0.03)	0.002	0.23 (0.06; 0.36)	< 0.001	0.36 (0.19; 0.49)	0.831	−0.03 (−0.12; 0.05)	< 0.001	0.28 (0.13; 0.46)	< 0.001	0.31 (0.19; 0.49)
Nitrogen concentration	0.078	2.15 (1.17; 3.05)	< 0.001	−3.95 (−5.12; −255)	< 0.001	−6.1 (−6.88; −4.8)	0.163	1.67 (−0.52; 3.67)	< 0.001	−5.55 (−7.6; −3.6)	< 0.001	−7.22 (−8.08; −6.45)
Nitrogen content	< 0.001	10.5 (8.22; 13)	0.265	−2.06 (−4.92; 0.87)	< 0.001	−12.6 (−15.1; −10.2)	< 0.001	9.87 (6.93; 11.9)	< 0.001	−10.8 (−12; −9.59)	< 0.001	−20.7 (−22.7; −17.9)
^15^Nitrogen recovery	-	-	-	-	< 0.001	−52.8 (−55.4; −50.4)	-	-	-	-	< 0.001	−61 (−62.8; −57.5)
Phosphorus concentration	0.933	−0.02 (−0.2; 0.08)	0.012	0.2 (0.1; 0.3)	0.006	0.23 (0.1; 0.39)	0.999	0.0 (−0.01; 0.01)	0.072	−0.15 (−0.23; −0.02)	0.067	−0.15 (−0.23; −0.02)
Phosphorus content	0.678	0.24 (−0.23; 0.57)	0.001	1.37 (0.69; 1.85)	0.003	1.13 (0.39; 1.74)	0.415	0.37 (−0.1; 0.84)	0.873	0.14 (−0.36; 0.78)	0.705	−0.23 (−0.56; 0.29)
N:P	0.052	2.03 (0.57; 4.33)	< 0.001	−4.63 (−5.88; −2.99)	< 0.001	−6.66 (−8.76; −5.06)	0.461	0.96 (−0.24; 2.08)	0.019	−2.43 (−3.83; −1.29)	0.002	−3.39 (−4.3; −2.81)
Root length	0.384	−5.25 (−8.75; −1.75)	< 0.001	21.5 (12.2; 30.8)	< 0.001	26.8 (17.2; 36)	0.920	1.5 (−5; 8.25)	0.001	19 (8.75; 29.5)	0.001	17.5 (7.25; 26.5)
Specific root length	0.228	5 (−0.25; 9.75)	0.003	11.8 (5.75; 17.5)	0.082	6.75 (2.5; 11.2)	0.228	5 (1; 11)	0.467	3.5 (0.5; 6.5)	0.864	−1.5 (−7.93; 2.25)

**Fig. 2:**
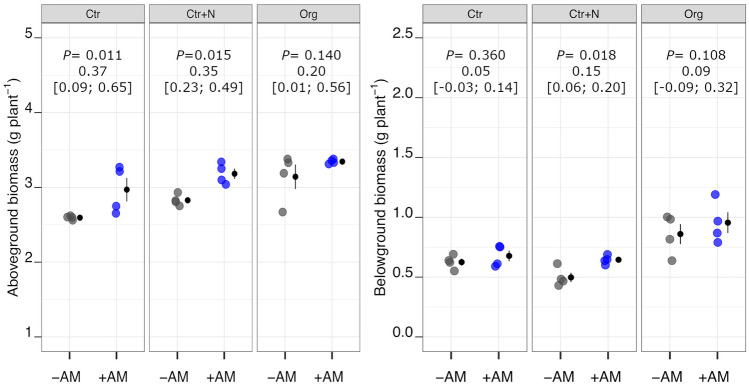
Aboveground and belowground biomass dry weight of durum wheat in the different fertilisation treatments (Ctr control not fertilised, Ctr+N control fertilised with ammonium sulphate, Org soil amended with crop residues) in the absence (grey points) or presence (coloured points) of arbuscular mycorrhizal (AM) fungal inoculum. Raw data are shown in the plot. Data are plotted with the mean depicted as a black circle ± SE (*n* = 4) represented by the end of the vertical black line to the right of the raw data. *p*-values for pairwise comparisons, effect size mean (unpaired means), and estimated 95% confidence intervals (CIs; in square brackets) between +AM and −AM conditions within the same fertilisation treatment are reported above the plots

**Fig. 3:**
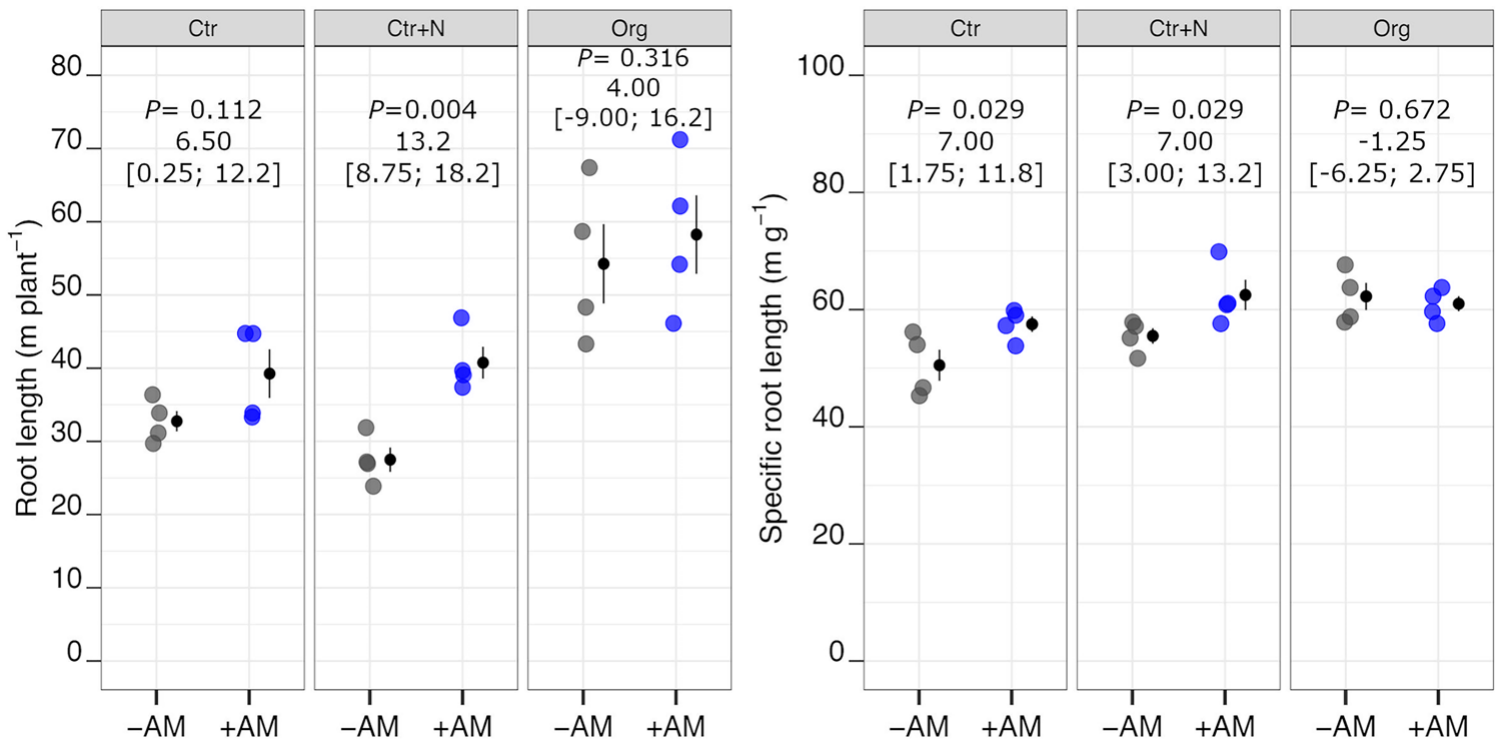
Root length and specific root length of durum wheat in the different fertilisation treatments (Ctr control not fertilised, Ctr+N control fertilised with ammonium sulphate, Org soil amended with crop residues) in the absence (grey points) or presence (coloured points) of arbuscular mycorrhizal (AM) fungal inoculum. Raw data are shown in the plot. Data are plotted with the mean depicted as a black circle ± SE (*n* = 4) represented by the end of the vertical black line to the right of the raw data. *p*-values for pairwise comparisons, effect size mean (unpaired means), and estimated 95% confidence intervals (CIs; in square brackets) between +AM and −AM conditions within the same fertilisation treatment are reported above the plots

On average, the +AM plants showed higher aboveground biomass than the −AM plants (+ 11%). The Org treatment increased shoot dry matter by 17% on average compared to Ctr treatment (Fig. 2), whereas, on the whole, no differences for this traits were observed between Org and Ctr+N nor between Ctr and Ctr+N (Table S1 and Table S2). On average, the +AM plants showed higher root dry matter and root length than the −AM plants (+ 15% and + 21%, respectively; Figs. 2 and 3). These two root traits were positively influenced by the addition of crop residues, both being higher in Org than Ctr and Ctr+N treatments (on average + 49% for root dry matter and + 60% for root length; Figs. 2 and 3; Table S2). Compared to the −AM plants, the +AM plants exhibited higher SRL (+ 7% on average). Both fertilisation with mineral fertiliser and crop residue amendment positively influenced SRL (on average + 9% and + 14%, compared to Ctr, respectively for Ctr+N and Org; Table S2).

**Fig. 4:**
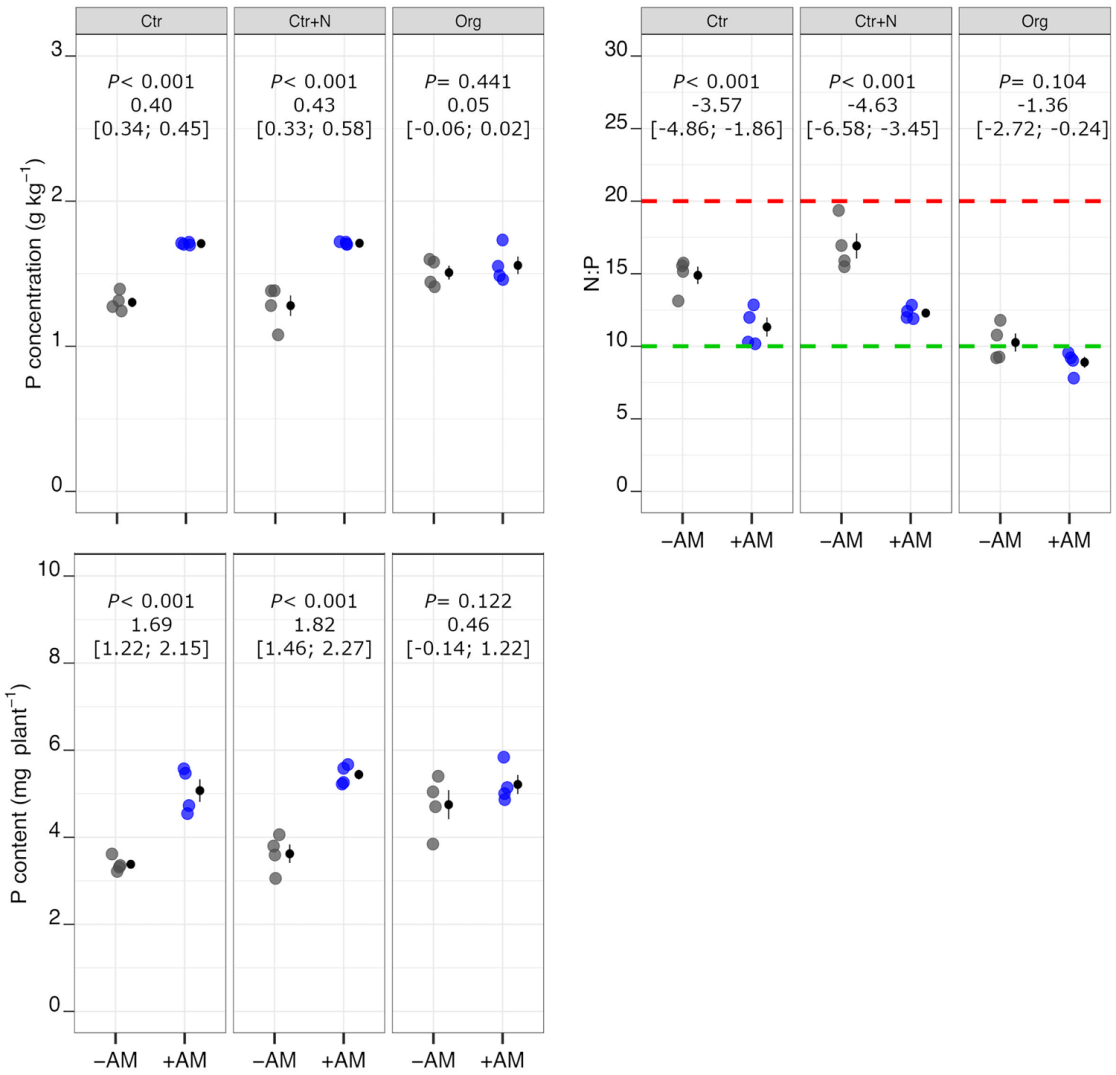
Phosphorous concentration and content, and N:P of durum wheat in the different fertilisation treatments (Ctr control not fertilised, Ctr+N control fertilised with ammonium sulphate, Org soil amended with crop residues) in the absence (grey points) or presence (coloured points) of arbuscular mycorrhizal (AM) fungal inoculum. Raw data are shown in the plot. Data are plotted with the mean depicted as a black circle ± SE (*n* = 4) represented by the end of the vertical black line to the right of the raw data. *p*-values for pairwise comparisons, effect size mean (unpaired means), and estimated 95% confidence intervals (CIs; in square brackets) between +AM and −AM conditions within the same fertilisation treatment are reported above the plots. Dashed lines in the N:P plot indicate plants growing in N-limiting (green) or in P-limiting (red) conditions according to Güsewell (2004)

**Fig. 5:**
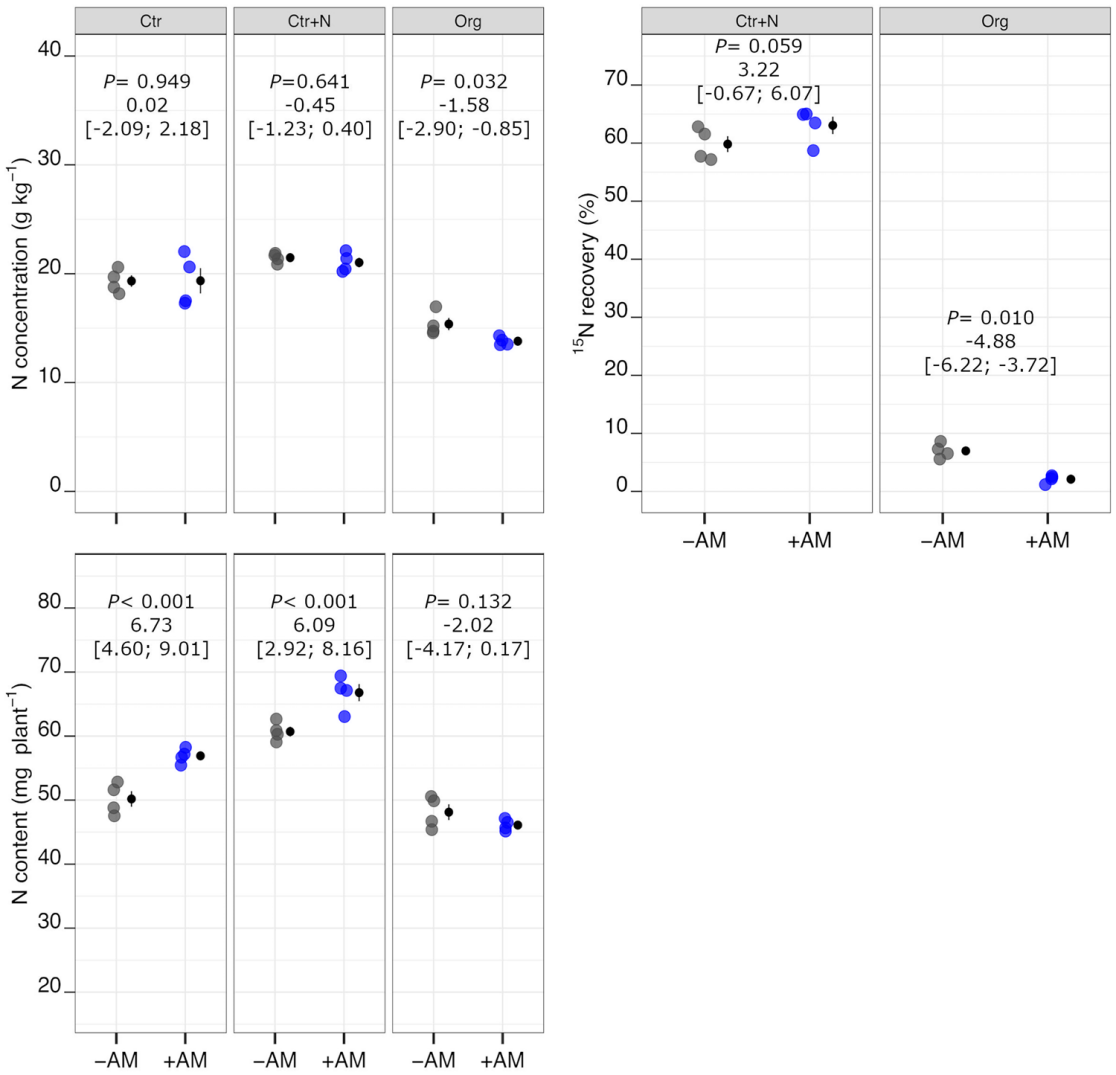
Nitrogen concentration and content, and ^15^N recovery from mineral fertiliser and organic matter of durum wheat in the different fertilisation treatments (Ctr control not fertilised, Ctr+N control fertilised with ammonium sulphate, Org soil amended with crop residues) in the absence (grey points) or presence (coloured points) of arbuscular mycorrhizal (AM) fungal inoculum. Raw data are shown in the plot. Data are plotted with the mean depicted as a black circle ± SE (*n* = 4) represented by the end of the vertical black line to the right of the raw data. *p*-values for pairwise comparisons, effect size mean (unpaired means), and estimated 95% confidence intervals (CIs; in square brackets) between +AM and −AM conditions within the same fertilisation treatment are reported above the plots

On the whole, AM symbiosis positively affected both P concentration and P content but the magnitude of this effect varied widely in relation to fertilisation treatment (Fig. 4); the increases being marked in Ctr and Ctr+N and modest in Org. Therefore, the effects of AM symbiosis on shoot growth appeared to a certain extent related to the variation of P concentration in shoot tissues and this supports the hypothesis that this element was a limiting factor. Consequently, in −AM plants, both P concentration and P content were higher in the Org treatment compared to both Ctr and Ctr+N treatments, whereas, when plants were inoculated with AM fungi, the differences among fertilisation treatments were very small or even null (Table 2). Plant N concentration was lower in +AM than −AM plants in the Org treatment, whereas no differences were observed with mycorrhization in Ctr and Ctr+N (Fig. 5). In the Org treatment, plant N concentration was markedly lower than in both controls (Ctr and Ctr+N). On the whole, N content was considerably affected by fertilisation treatment, decreasing in the order: Ctr+N > Ctr > Org (Table S2). AM symbiosis resulted in an increase of N content in Ctr and Ctr+N but not in the Org treatment (Fig. 5). On average, regardless of inoculation with AM fungi, the N:P ratio was markedly lower in Org compared to both Ctr and Ctr+N (Table S2). AM symbiosis determined a decrease in N:P ratio in Ctr and Ctr+N compared to the Org treatment (Fig. 4).

In the Ctr+N treatment, the percentage of ^15^N recovery was 61.5% on average, with no significant differences by mycorrhization (Fig. 5). The ^15^N recovery from added OM was extremely low (on average 4.5%) with strong differences between +AM and –AM plants (7.0% and 2.1%, respectively).

### Canonical discriminant analysis

**Fig. 6:**
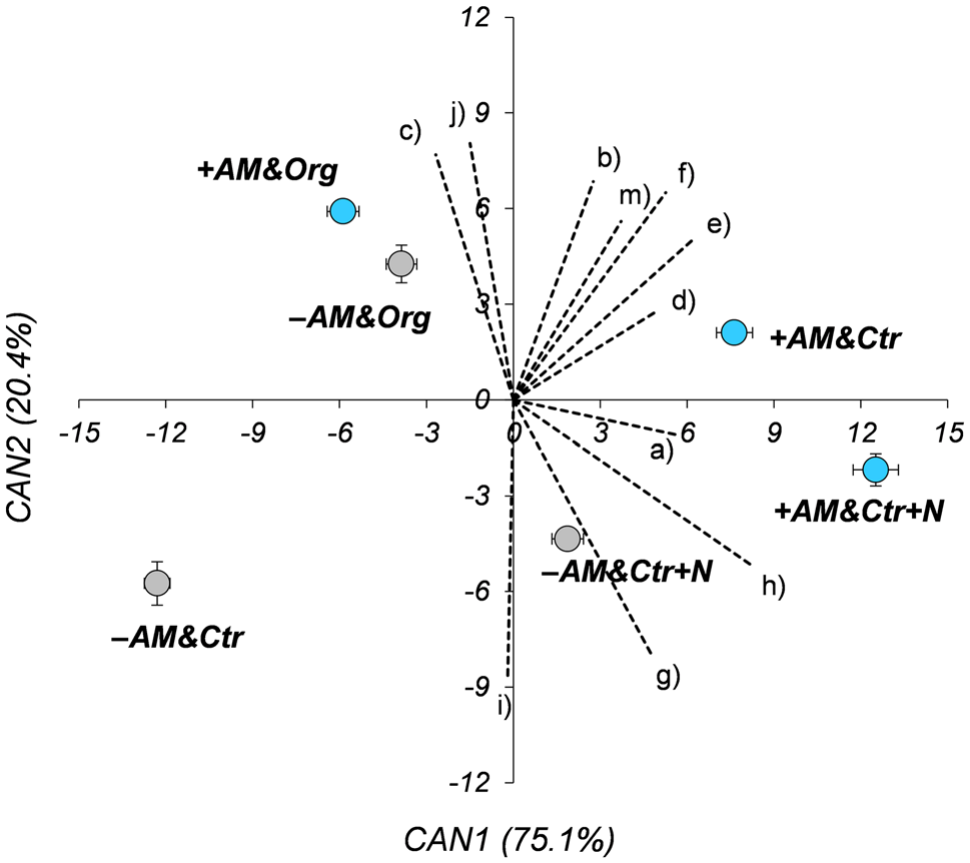
Canonical discriminant analysis (CDA). Canonical variable means (centroid values ± SEs) were calculated for each treatment (combinations of mycorrhization and fertilization factors). CAN1 first canonical variable, CAN2 second canonical variable. +AM, inoculation with AM fungi; −AM, non-inoculated. Ctr control not fertilised, Ctr+N control fertilised with ammonium sulphate, Org soil amended with organic matter. CDA was performed on the basis of 11 traits measured on plants: (a) number of tillers per plant; (b) shoot dry matter; (c) root dry matter; (d) leaf area; (e) P concentration; (f) P content; (g) N concentration; (h) N content; (i) N:P ratio; (j) root length; (k) specific root length

The CDA performed for all treatment combinations and based on all data clearly discriminated the treatments (Fig. 6). CAN1 accounted for 75.1% of the total variance and mostly varied according to N content (shown as ‘h’ in Fig. 6) and P concentration (shown as ‘e’ in Fig. 6), whereas CAN2 accounted for 20.4% of the total variance and was mostly influenced by N:P ratio, N concentration and root length (shown as ‘i, g, and j,’ respectively, in Fig. 6). +AM&Org and −AM&Org did not show a distance higher than the Mahalanobis squared distances (*p* = 0.398), whereas +AM&Ctr+N and −AM&Ctr+N did (*p* = 0.001). Additionally, +AM&Ctr and −AM&Ctr differed from each other by distance (*p* ≤ 0.001) and notably +AM&Ctr was projected in the hyperspace by the same vectors, direction, and magnitude of +AM&Ctr+N.

## Discussion

The present paper reports data from a pot study in which wheat plants were grown in a substrate low in P, in the presence or absence of AM fungal inoculum, and with the addition or not of a high C:N organic material (biomass of oats). Test conditions were intended to simulate a situation commonly encountered in Mediterranean (herbaceous) cropping systems, where wheat is undoubtedly a key crop, soils are generally poor in P available to plants, and crop residues of cereals often represent the sole source of OM that is returned to the soil. Our aim was to test whether inoculation with AM fungi could potentially enhance plant nutrient uptake and plant growth under these conditions. Briefly, our results did not show any relevant benefit of AM symbiosis to the host plant when a high C:N organic material was added to the P-limited substrate. Indeed, the addition of crop residues per se led to an increase of plant growth and P uptake and, under these circumstances, the benefits of AM symbiosis for plant P and N acquisition (and thus for plant growth) were constrained, most probably because of N becoming limiting as a result of an increased immobilisation of N by saprotrophic microorganisms stimulated by the presence of the AM fungi (or by retention of N from the added crop residues in external AM hyphae).

### Plant response to soil amendment with high C:N crop residues

In the absence of AM fungi, soil amendment with crop residues increased aboveground and belowground plant growth by 13.3% and 60.0%, respectively, as well as shoot P concentration and content (+ 17% and 36%, respectively) compared to the average of both Ctr and Ctr+N. Many authors have found that the addition of OM to a soil may enhance plant available P through various mechanisms including secretion of organic anions (mainly acids) by OM-decomposing microbes, which, at least in alkaline soils such as that of this experiment, causes a lowering of the soil pH in the rhizosphere and a concomitant enhancement of phosphate diffusion (Shen et al. 2011; Kovar and Claassen 2015); desorption of P from mineral surfaces by neutralising reaction sites that would normally fix P through ligand exchange reactions with inorganic or organic ligands (Richardson 2001; Guppy et al. 2005; Mackay et al. 2017); and release of phosphatase enzymes, which have a major role in organic phosphate solubilisation (Rodríguez et al. 1999; Richardson 2001). These aspects likely occurred in our conditions because of the high soil pH and low soil organic C content, which decrease P availability for plants and reduce soil microbial activity, respectively. In the present study, some of the experimental conditions imposed (soil maintained above 70% of the water holding capacity essentially over the entire duration of the experiment; oat biomass incorporated in soil after being chopped to very small pieces; temperatures quite high and constantly increasing over the experimental period) were certainly favourable for microbial growth and activity in the Org treatments, and consequently, for decomposition of the OM (both the added crop residues and the soil native OM). This probably enhanced and accelerated the biological cycling of P and thus increased the availability of P for plants, in accordance with Singh et al. (1988) who found, in a pot experiment, an increase (though small) in the P available to plants after only 4 weeks from soil incorporation of both rice and wheat straws.

That the advantages offered by the addition of crop residues are attributable largely to an increase in P availability and not that of N is evidenced by the addition of N (Ctr+N) having had modest effects compared to the not fertilised control (Ctr) and in any case, significantly less than those obtained with the addition of oat biomass (Org). Moreover, in the plants grown in the soil with crop residue amendment, both N concentration and N content were markedly lower in comparison to both controls; this suggests a marked decrease in the N available to the plants because of N immobilisation by an increased soil microbial biomass and activity, also taking into account the high C:N ratio of the OM applied. Soil microorganisms are indeed generally considered to be more effective than plants at competing for N in the short term (Hodge et al. 1998, 2000b; Owen and Jones 2001). In this experiment, stimulated soil microbial activity resulting from the addition of crop residues to the soil probably reduced the amount of N readily available for plant uptake and limited its accumulation in plant biomass but not so much as to negatively affect plant growth.

In light of this, in the Org treatment, the plants showed a markedly lower N:P ratio compared to both controls. Interestingly, a relationship seemed to emerge between the N:P ratio and the biomass allocation to roots and shoots, as already pointed out by other authors (Gryndler et al. 2002; De Groot et al. 2003; Güsewell 2004). The higher allocation of biomass to roots observed in the Org treatment might reflect lower N availability which could have stimulated plants to invest more in roots than in shoots to increase the chances of intercepting additional N by exploring a large volume of soil. At the same time, an increase in the availability of P induced by the addition of crop residues should have prompted the plant to reduce the biomass allocated to roots. Evidently, the effect of N was stronger than that of P, according to Andrews et al. (1999) and De Groot et al. (2003).

### Plant response to mycorrhization

Mycorrhization had positive effects on plant growth particularly in the control treatments (Ctr and Ctr+N). Here, the positive effects of AM symbiosis on plant growth appeared to be linked mainly to the increase in P uptake by the +AM plants compared to −AM plants. This result was expected because the substrate was poor in available P. Many studies report that in general the contribution of AM fungi to plant P uptake decreases with increasing soil P supply (Barea et al. 2008; Smith and Smith 2011a). Accordingly, the advantage of mycorrhization to plant P nutrition was great for Ctr and Ctr+N (+ 50% in the +AM compared to the −AM plants) which are where the P available for plant uptake was low. On the contrary, the advantage was small in Org (+ 10%), where plant available P increased after the addition of crop residues to the substrate. Compared to the non-mycorrhizal condition, AM symbiosis resulted in an increase of N content in both controls. According to Azcón et al. (2003), such a result likely depended on the increase of P availability for the plants in consequence of mycorrhization, P likely was the limiting factor for plant growth, and thus, mycorrhizal symbiosis, favouring greater P acquisition, stimulated plant growth and therefore, increased plant N demand.

In the Org treatment, mycorrhization, on the whole, produced null or modest effects on plant growth and plant nutrient uptake, so that the CDA showed no difference between −AM and +AM plants within the Org treatment. The greater availability of P resulting from the addition of crop residues to the soil made plants less dependent on mycorrhizal symbiosis. Under such conditions, plants should easily autonomously satisfy their P needs without expending photosynthates on mycorrhizae. Additionally, with regard to N uptake, mycorrhization in the Org treatment did not confer any benefit. The reason for this was probably that in the Org treatment, the presence of mycorrhizal hyphae stimulated soil microbial growth and activity (also by altering the structure of the soil microbial community in favour of those soil microorganisms that are responsible for decomposition of the added OM), as reported by other authors (Toljander et al. 2007; Hodge et al. 2010; Nuccio et al. 2013; Jansa et al. 2019). This in turn, considering the particularly high C:N ratio of the crop residues, amplified the immobilisation of N and thus, decreased N availability to plants, so that, at the end of the experiment, a lower plant N concentration was found in the Org+AM compared to −AM plants. Indeed, mycorrhization did not affect the overall plant N content in the Org treatment, although it strongly decreased the recovery of ^15^N from the added OM. Unfortunately, our data do not allow us to distinguish among the several possible mechanisms behind this result (the understanding of which would require knowledge of the timing with which N is made available from different sources, especially organic, and the removal rates by the different players who compete for N), but they suggest that the three players involved (plants, saprotrophic microbes, and AM fungi) probably exerted a differentiated competition for the different sources of N present in the substrate (mineral N and organic N from native or applied OM). Presumably, the N from the added crop residues has been used to a greater extent by the AM fungi and/or the saprophytic microbes (whose activity was likely stimulated by the presence of AM fungi) than by the plant, while the plant increased the direct uptake of inorganic ^14^ N from soil solution to boost its N acquisition as suggested by Saia et al. (2014), or preferentially received inorganic ^14^ N (as ammonium or nitrate) through translocation from the AM fungi. In a recent study, Klink et al. (2020) observed a higher ^15^N isotope natural abundance in the external AM hyphae of *Rhizophagus irregularis* relative to the leaf material of its host plant (*Festuca ovina* L.); thus, suggesting the occurrence of isotopic fractionation which could imply a preferential use of the ^15^N isotope by the AM fungus. Those authors suggested that ^15^N enrichment would indicate the potential of AM fungi to gain N from an organic source, despite translocation of ammonium and nitrate to the host plant. This could have occurred in our experiment.

It is interesting to note that the percentage of mycorrhization was not associated with a corresponding benefit in terms of plant growth and nutrient uptake. Indeed, mycorrhization was higher in the Org treatment than in the controls, whereas the opposite was observed in terms of benefits. Such contrasting results for the relationship between AM fungal root colonisation and its effects on plant performance have been reported (van der Heijden et al. 2006; Büscher et al. 2012; Fellbaum et al. 2014; Corrêa et al. 2015; Ingraffia et al. 2020). Notably, the mycorrhization percentage was measured at the end of the experiment and the edaphic conditions could have changed during the growing period; for instance, in the early stages of development, the temporary immobilisation of nutrients by decomposer microorganisms may have led to nutritional stress conditions, stimulating plants to activate the symbiotic relationship with AM fungi, which was followed by periods of greater mineral nutrient availability, as noted by Leigh et al. (2011).

In conclusion, the AM fungi did not help the plant in boosting its uptake of P and its growth when a high C:N organic material was added to the P-limited soil. Results from this study showed indeed that the addition of such organic material by itself increased plant P uptake and plant growth and that, under these circumstances, the benefits of AM symbiosis for plant P and N acquisition were limited, presumably because of the amplified immobilisation of N by decomposers stimulated by the presence of the AM fungi or by a retention of N taken up from the added OM in AM fungal extraradical mycelium. This suggests that the interplay of P and N availability from OM is potentially deterministic for the net benefits of arbuscular mycorrhizae to host plant performance. Further research is needed to fully circumscribe the conditions under which mycorrhizal symbiosis can play an effective role in mitigating the counterproductive effects of nutritional stresses in plants.

## Supplementary Information

Below is the link to the electronic supplementary material.

Supplementary file1 (DOCX 47 KB)
