# Supplementary material for: Addition of high C:N crop residues to a P-limited substrate constrains the benefits of arbuscular mycorrhizal symbiosis for wheat P and N nutrition
Source: Mycorrhiza. 2021 Apr 23;31(4):441–54. doi: 10.1007/s00572-021-01031-8 (PMC8266712; doi:10.1007/s00572-021-01031-8)
Supplement: Supplementary file 1 — Supplementary file1 (DOCX 47 KB) [file 572_2021_1031_MOESM1_ESM.docx]

**Addition of high C:N crop residues to a P-limited substrate constrains the benefits of arbuscular mycorrhizal symbiosis for wheat P and N nutrition**

**Mycorrhiza** (Electronic ISSN 1432-1890 – Print ISSN 0940-6360)

Rosolino Ingraffia^1a^, Sergio Saia^2a^, Antonio Giovino^3^, Gaetano Amato^1^, Giuseppe Badagliacca^4^, Dario Giambalvo^1^, Federico Martinelli^5^, Paolo Ruisi^1^*, Alfonso S. Frenda^1^

^1^ Università degli Studi di Palermo, Dipartimento di Scienze Agrarie, Alimentari e Forestali, Viale delle Scienze, 90128, Palermo, Italy

^2^ University of Pisa, Department of Veterinary Sciences, Via delle Piagge 2, 56124, Pisa, Italy

^3^ Council for Agricultural Research and Economics, Research Centre for Plant Protection and Certification (CREA-DC), SS 113 km 245.500, 90011, Bagheria (PA), Italy

^4^ Università Mediterranea di Reggio Calabria, Dipartimento di Agraria, Feo di Vito, 89124, Reggio Calabria, Italy

^5^ Università degli Studi di Firenze, Dipartimento di Biologia, Via Madonna del Piano 6, 50019, Sesto Fiorentino, Italy

^a^ Co-first authors

* Corresponding author: Paolo Ruisi. E-mail: [paolo.ruisi@unipa.it](mailto:paolo.ruisi@unipa.it)

**Fig. S1** Ten-day mean air temperature (blue) and 10-day maximum temperature (orange) during the experiment. The times of sowing (S), applications of the mineral N fertilizer (1F, 2F and 3F), and plant harvest (H) are indicated.

One-way ANOVA was used to determine the effects of fertilization on the observed traits. The analyses were performed with R version 4.0.2 (R Core Team 2020). Shapiro and Bartlett tests were used to assess normality and homoscedasticity, respectively, of the model residuals. Following the ANOVA, pairwise comparisons using the ‘emmeans’ package (Lenth et al. 2020) and effect size mean (unpaired means) and confidence intervals using the ‘dabestr’ package (Ho et al. 2019) were used to investigate differences between the three fertilization treatments. All *P*-values derived from selected pairwise comparisons and confidence intervals of the differences are reported in the tables.

**Tab. S1** Analysis of variance: *F*- and *P*-values for the effects of the fertilization treatment (Fert) on the traits observed in durum wheat plants. Data of each fertilization treatment includes pooled data of plants grown in the absence (–AM) or presence (+AM) of arbuscular mycorrhizal fungal inoculum

|  | Fert | | |
| --- | --- | --- | --- |
|  | *df* | *F*-values | *P*-values |
| Aboveground biomass | 2 | 6.82 | 0.006 |
| Belowground biomass | 2 | 27.77 | < 0.001 |
| Nitrogen concentration | 2 | 74.95 | < 0.001 |
| Nitrogen content | 2 | 45.82 | < 0.001 |
| ^15^Nitrogen recovery | 1 | 1315.19 | < 0.001 |
| Phosphorus concentration | 2 | 0.06 | 0.937 |
| Phosphorus content | 2 | 1.37 | 0.280 |
| N:P | 2 | 10.58 | < 0.001 |
| Root length | 2 | 23.74 | < 0.001 |
| Specific root length | 2 | 4.79 | 0.021 |

**Tab. S2** *P*-values for pairwise comparisons, effect size mean (unpaired means) and estimated 95% confidence intervals (𝚫 mean and CIs; in square brackets) in the different fertilization treatments (Ctr, control not fertilized; Ctr+N, control fertilized with ammonium sulphate; Org, soil amended with crop residues). Data of each fertilization treatment includes pooled data of plants grown in the absence (–AM) or presence (+AM) of arbuscular mycorrhizal fungal inoculum

|  | Ctr vs Ctr-N | |  | Ctr vs Org | |  | Ctr-N vs Org | |
| --- | --- | --- | --- | --- | --- | --- | --- | --- |
|  | *P*-values | 𝚫 mean ± 95% CIs |  | *P*-values | 𝚫 mean ± 95% CIs |  | *P*-values | 𝚫 mean ± 95% CIs |
| Aboveground biomass | 0.204 | 0.22 [-0.06; 0.42] |  | 0.004 | 0.46 [0.13; 0.6] |  | 0.164 | 0.24  [-0.02; 0.40] |
| Belowground biomass | 0.233 | -0.08 [-0.16; -0.01] |  | < 0.001 | 0.26 [0.13; 0.37] |  | <.0001 | 0.34 [0.21; 0.46] |
| Nitrogen concentration | 0.008 | 1.91 [0.65; 300] |  | < 0.001 | -4.75 [-6.06; -3.38] |  | < 0.001 | -6.66 [-7.38; -5.59] |
| Nitrogen content | < 0.001 | 10.2 [6.90; 13.9] |  | 0.005 | -6.44 [-9.06; -3.24] |  | < 0.001 | -16.6 [-19.5; -13.9] |
| ^15^Nitrogen recovery | - | - |  | - | - |  | < 0.001 | -56.9 [-59.6; -54.1] |
| Phosphorus concentration | 0.995 | -0.01 [-0.22; 0.19] |  | 0.965 | 0.03 [-0.12; 0.18] |  | 0.935 | 0.04 [-0.12; 0.22] |
| Phosphorus content | 0.787 | 0.30 [-0.66; 1.14] |  | 0.253 | 0.75 [-0.04; 1.42] |  | 0.598 | 0.45 [-0.28; 1.20] |
| N:P | 0.397 | 1.49 [-0.54; 3.98] |  | 0.015 | -3.53 [-5.04; -1.80] |  | 0.001 | -5.03 [-7.08; -3.27] |
| Root length | 0.859 | -1.88 [-8.62; 4.12] |  | < 0.001 | 20.2 [12.4; 27.7] |  | < 0.001 | 22.1 [13.8; 30.1] |
| Specific root length | 0.141 | 5.00 [0.50; 10.5] |  | 0.018 | 7.62 [3.75; 12.2] |  | 0.557 | 2.62 [-2.25; 6.12] |

**References**

Ho J, et al (2019) Moving beyond P values: Data analysis with estimation graphics. Nature Methods, 16(7), 565–566. https://doi.org/10.1038/s41592-019-0470-3

R Core Team 2020. R: A language and environment for statistical computing. R Foundation for Statistical Computing, Vienna, Austria. URL https://www.R-project.org/

Lenth R, et al (2020) emmeans: Estimated Marginal Means, aka Least-Squares Means. R package version 1.4.8. https://CRAN.R-project.org/package=emmeans
